# Prioritising maternal mental health and infant neurodevelopment research in Africa – A call for action amidst the backdrop of the COVID‑19 pandemic

**DOI:** 10.4102/sajpsychiatry.v28i0.1716

**Published:** 2022-01-18

**Authors:** Marlette Burger, Christa Einspieler, Marianne Unger, Dana Niehaus

**Affiliations:** 1 Department of Health and Rehabilitation Sciences, Faculty of Medicine and Health Sciences, Stellenbosch University, Cape Town, South Africa; 2 Research Unit (IDN) Interdisciplinary Developmental Neuroscience, Division of Phoniatrics, Medical University of Graz, Graz, Austria; 3 Department of Psychiatry, Faculty of Medicine and Health Sciences, Stellenbosch University, Cape Town, South Africa

## Introduction

Perinatal mental health is a major neglected public health concern in Africa. As a result of the pressures of communicable diseases and malnutrition, mental health services in Africa have always been poorly resourced. The coronavirus disease 2019 (COVID-19) caused a spiralling global pandemic; the collateral effects of COVID-19 may even cause greater inequalities in maternal mental health services in Africa. The first objective of this article is to provide a brief overview on the importance of the first 1000 days of life and the impact of maternal mental health disorders on nurturing care and the neurodevelopment of infants. The second objective is to advocate prioritising research on the impact of COVID-19 on maternal mental health and infant neurodevelopment in Africa.

Maternal mental health disorders are amongst the most common health problems during the perinatal period.^1^ Symptoms of maternal mental health disorders appear to escalate during and after pregnancy, particularly for women with a history of past psychiatric illnesses and women experiencing vulnerabilities such as socio-economic adversity, lower education, an unsupportive partner and poor family relationships.^2^ Since the turn of the 20th century, there has been an increase in research interest in the way maternal mental health shapes the foetus and the young infant’s early environment and influences later physical health and neurodevelopment. This article aims to review the reliance of infant neurodevelopment on maternal nurturing care and advocates prioritising maternal mental health and infant neurodevelopment research in Africa amidst the COVID‑19 pandemic.

## The importance of environmental influences and nurturing care during the first 1000 days of life

The first 1000 days of life, the period from conception and during full-term pregnancy (270 days) until the toddler’s second birthday (730 days), is seen as an essential window of opportunity, but also a time of great vulnerability, when the foundations for optimum neurodevelopment across the lifespan are established.^3^ The human central nervous system (CNS) is amongst the first and by far the most complex organ systems to begin its development prenatally and the last to complete it postnatally. Brain development continues well into the third decade when it reaches adult maturity, but the first two years of life are the most dynamic and fundamental phase of postnatal brain development. The rapid structural and functional brain development and the significant increase in total brain volume suggest that the first 1000 days of life is a critical period in which early life experiences lay the foundations of neural architecture. No other period of human life will carry out such a profound transformation in such a short period of time, and many of the neurodevelopmental changes expected to take place during the first 1000 days of life will not be able to occur in later life.^4^ Early neurodevelopment refers to the gradual and protracted unfolding of a child’s sensory-motor, cognitive, language, behaviour and social-emotional capacities shaped and influenced by a wide range of ongoing, dynamic, inextricable interactions between the environment, experience, and genetics.^5^

Prenatal development is primarily driven by genetic processes, although environmental factors, such as exposure to maternal stress hormones or illnesses, nutritional deficiencies, and teratogens may adversely shape prenatal brain development.^6,7^ Genetic programming provides the basic blueprint for the brain’s fundamental architecture; however, it is the environment that shapes the child’s developmental trajectory.^4,7,8^ Hence, environmental conditions and experiences profoundly affect the postnatal development of the brain.^7^ In the presence of a sensitive and responsive primary caregiver, healthy nutrition and a supportive, stable and safe home environment, the brain typically thrives.^4,7^

Compared to other mammalian species, one of the unique characteristics of the human infant is their prolonged period of extreme dependence on an adult caregiver for the provision of nutrition, shelter, safety, and comfort (emotional and physical). Therefore, the parent-infant caregiving relationship is the earliest, most meaningful, profound and enduring experience of infancy and childhood.^9^ Regardless of the primary caregiver’s identity, healthy child development can only take place in the context of a close and long-term dependable nurturing relationship. In fact, nurturing care is what the child’s brain requires and relies upon for optimal neurodevelopment.^8,9^ Nurturing care offers opportunities for early learning and interactions that are sensitive, responsive, emotionally supportive, developmentally stimulating and appropriate.^8,9^ Therefore, nurturing care promotes optimal physical, psychological, social-emotional, linguistic, and cognitive development. The World Health Organization (WHO) has stated that the quality of early caregiving interactions is one of the most significant modifiable environmental factors contributing to child development, especially in high-risk populations.^10^

Children living in poverty are at a greater risk for delayed cognitive, language, social-emotional and behavioural development that can influence their later academic achievement and contribute to lifetime-reduced occupational attainment.^11^ Healthy caregiver–child interactions mitigate the detrimental effects of low socio-economic status on the brain and are shown to safeguard against the negative impact of poverty and environmental adversities.^10,12^ In the framework of these early nurturing caregiver–infant interactions, the child has a strong biological drive to form a secure, emotional attachment to the primary caregiver. Early neurodevelopment is a complicated, bidirectional process. Subsequently, nurturing and responsive caregiving and secure attachment are associated with enhanced infant and child physical and mental well-being, emotional and behavioural resilience, better academic achievement, productive employment and less involvement with crime and violence in adulthood.^9,13^ In fact, healthy child development is seen as the cornerstone of a productive society with a prosperous and sustainable future.^13^ Accordingly, there is perhaps nothing more important a mother can do than foster a healthy and nurturing maternal-child caregiving relationship. Nurturing care is even more important if mother-infant pairs live in challenging socio-economic environments.

## Maternal mental health – Why does it matter?

‘There is no such thing as a baby … if you set out to describe a baby, you will find you are describing a baby and someone (p. 88).’^14^ With this statement, the English paediatrician and psychoanalyst Donald Winnicot (1952) emphasised the concept that the well-being of an infant and mother are intimately entwined during pregnancy and the first postnatal months. The foetus and infant directly experience the mother’s life. Consequently, their well-being and development are shaped by the mother’s environmental experiences and physical and mental health.^9^ A healthy, competent, and emotionally available mother contributes to the programming of a child’s healthy growth and development, and lays the groundwork for well-being. Motherhood, however, is a major event with enormous demands on the physical, psychological, social, and biological domains of a woman’s life. Therefore, the perinatal period may be even more challenging for women who suffer from mental health disorders.^9^

Common mental health disorders during the perinatal period include major depression and anxiety disorders, but it may also include postpartum psychosis, and post-traumatic stress disorder (PTSD).^15^ Symptoms of mental health disorders such as loss of interest in caring for oneself and others, tiredness, sleep disturbances, loss of motivation and energy, irritability and/or hostility, restlessness, lack of focused attention, slowed thinking, judgement and movement, hopelessness and preoccupation with worries and anxiety can severely compromise maternal caregiving behaviours.^10,16^ A mother is required to constantly adapt to a growing infant’s changing needs. The mother with a mental health disorder may be less sensitive to her child’s needs, and she may be unable to respond consistently and appropriately. Under these debilitating circumstances, she may not be able to create a nurturing caregiving environment for her child. Hence, her ability to provide healthy nutrition, safety, empathy, love and warmth, emotional regulation, predictable social engagement, cognitive stimulation and early learning opportunities may be severely impaired.^10,16^

Africa bears a disproportionate burden of disease and poverty, and the mother’s ability to provide and maintain nurturing care and form secure attachments may be further compounded by high levels of socio-economic adversity.^10,16^ Diseases such as malaria, HIV infection and acquired immunodeficiency syndrome (AIDS), as well as tuberculosis (TB) are common amongst women of childbearing age.^17,18,19^ Environmental hardships include unemployment, low education, poverty, lack of adequate nutrition and clean running water, poor sanitation, exposure to violence, including intimate partner violence, alcohol and drugs, and single parenthood.^12,16,17^ Single- and female-headed house-holds have become the norm in many parts of Africa^20^ with 42% of African children living in female-headed house-holds.^21^ As the whereabouts or even identity of many of these absent fathers are unknown,^21^ the caregiving and financial provision become the responsibility of the mother and/or grandmother sharing the house-hold. In these challenging circumstances, the mother has to provide for financial needs, practice self-care, be sensitive and responsive to her child’s physical and emotional needs, provide adequate nutrition and obtain appropriate healthcare for the child, for example, regular Well-baby clinic visits and immunisations. Undeniably, mothers with mental health disorders living in Africa face huge burdens. Their ability to establish nurturing relationships and lay the foundation for their child’s healthy life-course physical and neurodevelopmental trajectories may therefore be seriously compromised.

## What do we know about the effect of maternal mental health disorders on infant development?

A large body of evidence emanating from a small proportion of highly developed, resource-rich countries suggests that maternal mental health disorders may, directly and indirectly, influence the child’s development. Numerous systematic reviews conducted in high-income countries (HICs) have established associations between pre- and postnatal exposure to maternal psychiatric disorders and adverse infant and/or child neurodevelopment.^16,22,23,24,25,26^ A recent systematic review by Burger et al. (2020) analysed the evidence for perinatal mental health disorders and their association with infant and toddler neurodevelopment during the first two postnatal years.^27^ This study was unique since it was the first systematic review that exclusively focused on the data of studies conducted in low-income, lower-middle-income and upper-middle-income economies as classified by the World Bank.^28^ During the initial search, the authors identified potential titles and abstracts from both HICs and low- and middle-income countries (LMICs). The literature search revealed that the vast majority of studies on this topic, published during the past three decades, were conducted in HICs. Only 24 studies were eligible for inclusion in the systematic review, which was alarmingly low when considering that almost 90% of the world’s children live in LMICs.^29^ Of the 24 included studies, only five were conducted in Africa. Contrary to evidence from systematic reviews generated in HICs, no clear associations between different types of pre- and postnatal mental health disorders (e.g. depression, anxiety and/or posttraumatic stress disorder) and infant or toddler motor, cognitive, language, behaviour and social-emotional development were found.^27^ Possible explanations for the lack of clear associations between perinatal mental health disorders and infant neurodevelopment in LMICs may be because of the fact that many additional factors (e.g. quality of parenting and home environment, maternal education, partner or social support and socio-economic status) may act as mediating or moderating factors and change the strength of associations between maternal mental health and child development.^16^ Mother-child dyads in LMICs are exposed to multiple risk factors and pathways between maternal mental health and child development are still poorly understood.^27^ Furthermore, the majority of studies used outcome measures to assess neurodevelopment that were validated in HICs, but not necessarily in LMICs where the studies were conducted.^27^ However, since the development of norm-referenced tools in LMICs is not always financially feasible, researchers should also include healthy mother-child dyads as a local reference group.^27^ It is evident that we still do not know how the neurodevelopment of infants and toddlers living in LMICs, and especially in Africa, are affected by maternal mental health disorders, and therefore further research is needed.

## The 10/90 knowledge gap – An urgent call for action

This knowledge gap on maternal mental health and infant neurodevelopment in Africa is part of the 10/90 divide in mental health research, which was identified by the turn of the 20th century.^30^ The 10/90 divide is where only 10% of research addresses the health-related problems of 90% of the global population living in resource-constrained settings.^31^ Over the last decade, researchers have become increasingly concerned with the 10/90 gap, particularly in the field of maternal mental health and child development. An expert statement on maternal mental health and child health and development in LMICs by the WHO and the United Nations Population Fund (UNFPA) concluded that there is a paucity of scientific data on the impact of maternal mental health disorders on child development in resource-constrained settings.^32^ They advised, that it is imperative for LMICs to generate evidence concerning the prevalence and adverse effects of maternal mental health disorders.^32^ The lack of data on perinatal mental health and child neurodevelopment in LMICs contributes to the invisibility and insignificance of burdens in these domains and can be summed up as follows: ‘no data – no problem – no action’ (Alfredo Solari, quoted by Tinajero, Cohen and Ametorwo, 2016; page 122).^33^

## The coronavirus disease 2019: The impact on perinatal mental health

The COVID-19 pandemic has created new challenges for pregnant and postpartum women and their infants living in LMICs. Pre-pandemic data revealed that the prevalence of perinatal health disorders in LMICs was substantially higher compared to rates reported in HICs. A meta-analysis of the prevalence of non-psychotic perinatal mental health disorders in low- and lower-middle-income countries indicated mean prevalence rates of 15.6% during pregnancy and 19.8% postnatally.^2^ This was higher compared to rates reported in HICs, namely 10% during pregnancy and 13% postnatally.^2^ A recent systematic review and meta-analysis reported that the prevalence of antenatal anxiety was significantly higher in LMICs (34.4%) compared with HICs (19.4%), while the prevalence of postnatal anxiety across the first 6 months postpartum was also significantly higher in LMICs (25.9%) compared with HICs (13.7%).^34^ Furthermore, the COVID-19 pandemic has taken an unprecedented toll on all health services around the globe, and maternal mental health has been bearing its brunt.

New evidence about the impact of COVID-19 on the perinatal mental health status of women living in LMICs is gradually emerging. A cross-sectional survey conducted in February 2020, during the peak of the COVID-19 epidemic in China, reported prevalence rates of 89.1%, 18.1%, and 45.9%, respectively, for perceived stress, anxiety, and depression amongst pregnant women.^35^ In Brazil, 40.5% of mothers reported moderate to severe stress, 25.9% suffered from anxiety, and 29.3% were depressed during the first 12 months postpartum because of the current COVID-19 pandemic.^36^ It is already evident that the psychological, social and economic effects of the COVID-19 pandemic are pervasive and escalating. The higher risk of maternal mental health disorders might be related to the impact of the pandemic on socio-economic circumstances, such as loss of employment, financial hardship, and food insecurity. Psychosocial stressors faced by mothers living in LMICs include isolation from social and family support networks and limited or no access to healthcare facilities as a result of lockdown regulations. Furthermore, anxiety about contracting COVID-19 and mother-to-foetus or infant transmission, increase in intimate partner violence, and bereavement from COVID-19 deaths may also have a major impact on perinatal mental health.^36,37,38^ At the time of writing, no evidence on the perinatal mental health status of women living in Africa during the COVID-19 pandemic is available, but there is a real risk that the impact may be similar or even higher than reported in China^35^ or Brazil.^36^

## The coronavirus disease 2019 and beyond – The need to generate knowledge on perinatal mental health in Africa

As a result of the global COVID-19 pandemic, there is even a more pressing need for further research to assess the impact of this pandemic on the perinatal mental health of women and the development of their offspring.^35^ Members of the ‘Research Innovation and Sustainable Pan-European Network in Peripartum Depression Disorder – Riseup-PPD’ team established the ‘Perinatal Mental Health and COVID-19 Pandemic’ task force. They are currently embarking on a large international cohort study in 11 European countries and three countries in South America to investigate the impact of the COVID-19 pandemic on perinatal mental health.^39^ The African region has a shortage of mental health resources, medical professionals and infrastructure, and the health systems are highly vulnerable during a pandemic.^40^ Therefore, there is an urgent need for research teams to explore how the COVID-19 pandemic intersects with perinatal mental health in Africa. The current preliminary findings of the heightened risk for perinatal mental disorders during the COVID-19 pandemic in LMICs,^35,36^ accompanied by the knowledge of the detrimental impact of poor perinatal mental health on child neurodevelopment in HICs, underscore the prioritisation of perinatal mental health research in Africa. In fact, the COVID-19 pandemic may even provide unique opportunities to address the historic underinvestment in maternal and child health research in Africa.^41^ Knowledge will not only aid in recognising the extent of maternal mental illnesses and how it affects infant development, but it will also help convince international stakeholders to allocate the necessary funding and resources towards maternal mental and child healthcare. Furthermore, understanding the early rearing environment of young African children amidst the COVID-19 pandemic is important. Failure to address maternal mental health problems may have far-reaching consequences over the child’s life course and into the next generation, long after the COVID-19 pandemic resolves.^42^

## Conclusion

Compelling scientific evidence confirms the tremendous importance of the first 1000 days for human development and the enduring detrimental effects of perinatal mental health disorders on infant and child neurodevelopment. This paper sought to highlight that because of the COVID-19 pandemic and the scarcity of health resources in Africa, knowledge of perinatal mental health and child neurodevelopment is paramount. Although it is necessary for scientists, clinicians, and health authorities across the globe to focus on the current COVID-19 pandemic, neglecting maternal mental health and child development in Africa will have far-reaching consequences. The coronavirus disease 2019 should not be fought at the expense of other diseases. In summary, maternal mental health and infant development in Africa must be guarded through research, advocacy, and practice.^41^
